# Riverine woodlands as a dynamic source of the marine sedimentary carbon sink

**DOI:** 10.1093/pnasnexus/pgag229

**Published:** 2026-06-25

**Authors:** Ulrike Herzschuh, Josefine Friederike Weiß, Laura Schild, Ingolf Kühn, Marie-Luise Kapsch, Heike H Zimmermann, Kathleen R Stoof-Leichsenring, Dirk Sachse, Dietrich Borchardt

**Affiliations:** Helmholtz Centre for Polar and Marine Research, Research Unit Potsdam, Alfred-Wegener-Institute (AWI), Telegrafenberg, 14473 Potsdam, Germany; Institute of Environmental Science and Geography, University of Potsdam, 14469 Potsdam-Golm, Germany; Institute of Biology and Biochemistry, University of Potsdam, 14469 Potsdam-Golm, Germany; Helmholtz Centre for Polar and Marine Research, Research Unit Potsdam, Alfred-Wegener-Institute (AWI), Telegrafenberg, 14473 Potsdam, Germany; Helmholtz Centre for Polar and Marine Research, Research Unit Potsdam, Alfred-Wegener-Institute (AWI), Telegrafenberg, 14473 Potsdam, Germany; Department Community Ecology, Helmholtz Centre for Environmental Research—UFZ, 06120 Halle, Germany; Max Planck Institute for Meteorology, Ocean in the Earth System (OES), 20146 Hamburg, Germany; Glaciology and Climate Department, Geological Survey of Denmark and Greenland (GEUS), 1350 Copenhagen, Denmark; Helmholtz Centre for Polar and Marine Research, Research Unit Potsdam, Alfred-Wegener-Institute (AWI), Telegrafenberg, 14473 Potsdam, Germany; GFZ Helmholtz Centre for Geosciences, Section 4.6 Geomorphology, Telegrafenberg, 14473 Potsdam, Germany; Geography Department, Humboldt Universität zu Berlin, 12489 Berlin, Germany; Department of Aquatic Ecosystem Analysis, UFZ Helmholtz-Centre for Environmental Research, 39114 Magdeburg, Germany

## Abstract

Land plants account for a significant proportion of the organic matter found in marine sediments. However, due to methodological limitations, the specific ecosystems and taxa contributing to this carbon sink have largely remained unknown. By leveraging ancient DNA data from marine sediment cores, this perspective article identifies riverine woodlands as key sources of marine sedimentary organic matter. These highly productive ecosystems grow directly along the land-coastal-ocean carbon transport pathway that links the short-term biological with the long-term geological carbon cycle. Furthermore, they are characterized by taxa that are resistant to decay, such as willows in mid-to-high northern latitudes and mangroves in tropical regions. We hypothesize that at the end of the last glaciation, a major negative feedback loop was established, where warmer temperatures caused glacial melt and increased runoff, promoting riverine vegetation growth. This, in turn, enhanced carbon transport into marine sediments, reduced atmospheric CO_2_ levels, and ultimately contributed to a cooling effect. The efficiency of organic matter burial was further amplified by the increased mineral supply and high sedimentation rates resulting from the mobilization of widespread unconsolidated glacial sediments. Pollen records and other data suggest that human land use led to the widespread disturbance of riverine woodlands globally in the last few hundred years, likely disrupting this feedback loop under current warming conditions. A comprehensive understanding of the interactions between climate, land use, riverine woodland loss, and marine carbon sinks is urgently needed to guide conservation strategies that aim to enhance this natural carbon capture and storage mechanism.

## Riverine woodlands in the land–ocean carbon continuum

Land plants account for a significant proportion of the organic matter found in marine sediments (1). Estimates suggest that about a third of organic matter in shelf and coastal marine sediments is of terrestrial origin (2) including biospheric (recent vascular plant detritus), soil organic carbon (OC), and petrogenic OC derived from erosion of sedimentary rocks (3). These sediments act as a major long-term sink for atmospheric CO_2_ (3, 4). Still, the source ecosystems and major contributing taxa are largely unknown.

Only a relatively small fraction of terrestrial primary productivity is transferred to sediments (5) predominantly through fluvial pathways (6). Here, photosynthetically fixed land plant carbon is, at least partly, converted into stable organo-mineral associations, eroded, transformed, and (re)deposited (7), such that river watersheds and floodplains act as important reactors of terrestrial organic matter that enable its stabilization. On a global scale, riverine particulate organic matter mainly derives from watersheds in the high northern and tropical latitudes (8).

Fluvial landscapes are often dominated by highly productive riverine woodlands (9–11) (Box 1) such as those formed by willow (*Salix*) in the mid-to-high northern latitudes (9) and by mangroves (mainly Rhizophoraceae) in the tropics (Box 1). Notably, despite covering <4% of the global land area (global wetland outlook—15), riverine woodlands may contribute a significant proportion of sedimentary organic matter to the ocean. This may be due to specific traits of riverine woodland plants, such as their biomass-rich rooting systems, which enhance their ability to grow under low oxygen conditions, to overcome sedimentation when flooded, and to grow directly in river channels (16). In addition, specific antimicrobial compounds, such as phenolic glycosides like salicin in *Salix* bark and leaves (17) and compounds supporting physical shielding, like lignin, make their tissue particularly resistant to decay (18). These factors may contribute to the overrepresentation of organic matter from riverine woodlands compared to other terrestrial ecosystems and taxonomic groups in sediments such as upland forest trees. This is consistent with the observation that outgassing of CO_2_ from rivers mainly derives from riverine wetland plants (19). However, despite their high potential as a carbon source the contribution of riverine woodlands to the marine sedimentary carbon sink is unknown.

Box 1 Riverine woodlands and mangroves. Wetlands are among the most diverse and productive ecosystems on Earth. Riverine woodlands are located along the flowing waters of rivers and streams (as opposed to bog and swamp forests on standing water) (9) and experience periodic flooding, erosion, and sediment accumulation (12).

The composition of riverine woodlands varies significantly along the length of a river, across its banks and valleys, and with changes in latitude. In the arctic and alpine zones, riverine woodlands are predominantly formed by willow shrubs (*Salix* spp.), which are joined by tree species such as alder (*Alnus* spp.) and birch (*Betula* spp.) at lower elevations and in subarctic regions. In midlatitude lowland floodplains, a zonation is often observed, where willow-dominated woodlands are found close to the permanent riverbed, transitioning into less frequently flooded and more diverse woodlands at the floodplain margins, with species like lime (*Tilia* spp.), ash (*Fraxinus* spp.), and oak (*Quercus* spp.). In tropical estuarine regions, such as the Orinoco Delta in northern South America, riverine woodlands take the form of mangrove forests, which are composed of salt-tolerant trees and shrubs. Key species in these ecosystems belong to the Rhizophoraceae family, which includes genera such as *Rhizophora* and *Bruguiera*.

The permanent water and nutrient supply make riverine woodlands highly productive ecosystems. Their massive stands slow down the water flow, which facilitates sedimentation along riverbanks. The burial of plant litter under mineral sediments aids in the formation of stable organo-mineral compounds which eventually become remobilized and transported along riverine pathways over thousands of years (13). The direct connection between woodlands with rivers ultimately enhances the transfer of carbon to long-term oceanic carbon sinks.

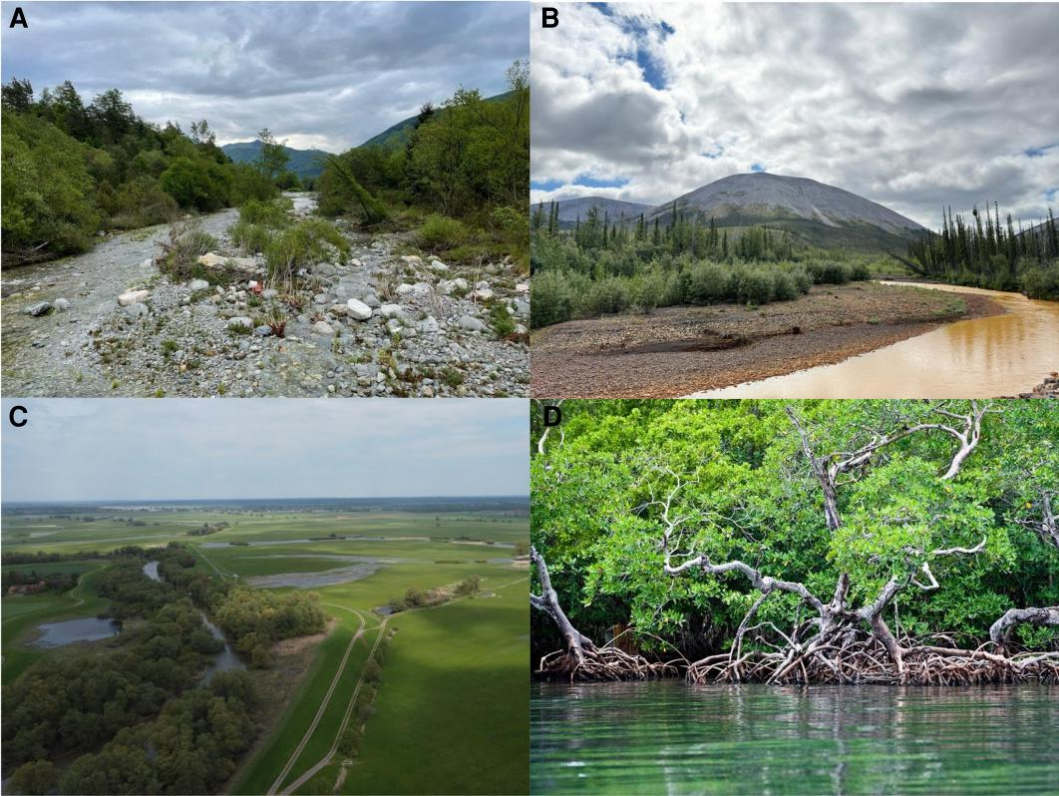

A) Frequently flooded riverine woods in Santa Maria Maggiore, Piemont, Italy (© Ingolf Kühn 2023). B) Riverine *Salix* vegetation in Alaska, USA (© Ulrike Herzschuh 2024 (14)). C) Drone picture of a modified fluvial landscape of the Elbe floodplain (© Dirk Sachse). D) Mangrove trees along the coast of Belize, South America (© Antonio Busiello/WWF).

Riverine woodlands are highly sensitive to fluctuations in climate and runoff (20) and have been considerably disturbed by anthropogenic land use (21, 22) which has modified their sedimentary carbon sink capacity to an uncertain degree. Our perspective article emphasizes that uncertainties about riverine woodlands’ contributions to marine sediments limit our ability to mitigate climate and human impacts on carbon sinks. We assess the potential of sedimentary ancient DNA (sedaDNA), an emerging technique with the power to determine the taxonomic composition of land plants from organic matter and ultimately to determine the role of riverine woodlands in the marine sedimentary carbon sink. Additionally, we explore how the dynamics of this sink have evolved in response to the warming at the end of the last glacial period and speculate how land use changes, later on, may have disturbed the natural source-sink-links. These insights hold implications for prioritizing area protection and devising strategies for natural carbon capture approaches.

## sedaDNA—a new opportunity to trace the terrestrial carbon source in marine sediments

**Figure 1:**
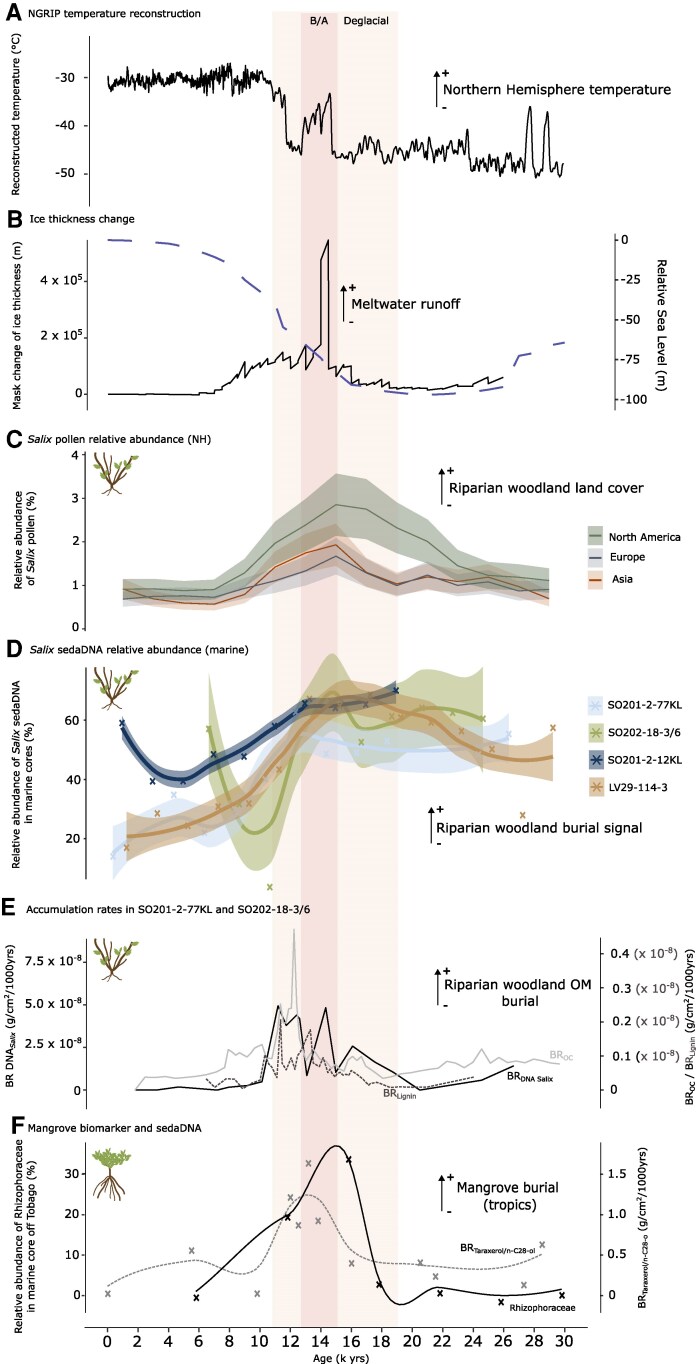
Contribution of riverine woodlands to organic matter in marine sediments from the northern North Pacific during the last 30,000 years, reflecting on-land woodland expansion peaking during the Bølling–Allerød in response to a warming and runoff strengthening from decaying ice sheets and glaciers. A) Temperature change as derived from reconstructions of the NGRIP ice core during the last 30,000 years (30). B) Freshwater release from the Northern Hemispheric ice sheets, indicating an increased meltwater run-off during the peak of the deglaciation. Values are obtained as changes in the ice-sheet thickness from the ICE-6G ice-sheet reconstructions (31, 32). The blue dashed line shows relative sea level change over the last 30,000 years (33). C) Riparian wetland changes as indicated by mean *Salix* percentage change in Northern Hemisphere pollen records from terrestrial archives (2,956 sites). D) Percentage of *Salix* reads (loess smoothed) relative to all land plant reads over the last 30,000 years retrieved by metagenomic analyses of four marine sediment cores from the northern North Pacific shelf regions (14, 34, 35). *Salix* abundance peaks during the deglacial and declines toward the Holocene. E) BR of *Salix* DNA reads of the marine sediment core SO201-2-77KL from the Shirshov ridge (North Pacific) showing highest values during the deglacial, particularly during the Bølling–Allerød string warming phase. This is generally in line with the lignin biomarker and total organic matter accumulation rate from the same sediment core (27, 28). F) Changes of mangrove to marine organic matter burial (loess smoothed) as indicated by Rhizophoraceae reads relative to all land plant reads over the last 30,000 years retrieved by metagenomic analyses of marine sediment core from Tobago basin (M78/1-235-1) (14) and the accumulation rate of a mangrove lipid biomarker from a close location (36).

The knowledge gap regarding the contribution of riverine woodlands to marine sedimentary carbon sinks primarily stems from methodological limitations in identifying the source taxa of organic matter in marine sediments with sufficient taxonomic resolution (23). Current state-of-the-art organic geochemical and isotope proxies provide basic information on bulk organic matter, enabling a rough distinction between marine and terrestrial sources, as well as petrogenic, soil, and biospheric origins (24–28). Furthermore, land plant-specific molecules offer broad insights into plant types (23). However, leaf waxes indicating broadleaf vegetation (29), lignin as a general biomarker for woody taxa, and taraxerol as a mangrove biomarker still represent the highest resolution proxies used for tracing riverine woodlands to date (Fig. 1; 37), but they rarely offer species-specific insights (38).

For decades it has been known that marine sediments contain intra- and extracellular DNA and that this is linked to the land DNA pool via river discharge (39). Furthermore, the analysis of sedaDNA is now routinely used to trace past changes in land ecosystems (40–42) and since recently also marine ecosystem changes are investigated (34, 35, 43). This is primarily due to the ability to identify taxa with a high degree of taxonomic precision.

As a carbon-source tracer, sedaDNA most directly fingerprint matter derived from recently living organisms (vegetation and surface soils) and does not capture petrogenic (“fossil”) OC derived from sedimentary rocks. Environmental DNA enters soils and waters as intra- and extracellular DNA from plant tissues (39, 44) and is transported via the two primary pathways responsible for fluvial OC export: as discrete particles and as mineral-associated OC (MAOC; 7, 13). While the relative proportions of these pathways vary depending on catchment size and climatic conditions, studies in subtropical systems have shown that MAOC can account for 55–78% of total OC export, with its importance increasing downstream due to floodplain connectivity (7). Accordingly, DNA can be transported dissolved, in particulate form, or attached to suspended mineral surfaces (39, 45).

Thus key advantage of sedaDNA, as opposed to other plant proxy data, stems from the fact that the signal comes directly from vegetative structures such as roots and leaves that form the primary biomass (41). This matter is then transported by runoff and erosion to the sedimentary basin, unlike generative elements such as pollen (or certain seeds) that can be widely dispersed by air (46). Preservation is facilitated by adsorption onto mineral surfaces and by physical shielding within recalcitrant cell walls, whereas warm and oxic conditions accelerate fragmentation and loss (18, 41, 45). This hydrodynamic sorting and differential stabilization of fresh plant debris versus MAOC—processes likely transferable across river systems (7)—implies that DNA signals are subject to the same transport biases as the bulk OC pool. Furthermore, lateral sediment transport and bioturbation can smear the timing of source signals (39, 41).

Despite this taphonomic advantage of sedaDNA, hitherto paleogenetic studies, even from land archives, almost exclusively address ecological questions, while tracing the source of sedimentary OC has rarely been the focus (eg for permafrost; 47). Studies comparing modern sedimentary eDNA from high-latitude and high-altitude lakes with surrounding vegetation find a strong overrepresentation of wetland taxa compared to upland vegetation, which was explained by the taphonomic peculiarity of sedimentary DNA to be transported by runoff (8, 46, 48). However, these terrestrial studies were applied using plant metabarcoding which provides only a semiquantitative signal of the quantitative plant composition. This is mainly due to biases originating from PCR amplification steps in the laboratory protocols and does not allow for the quantification of the sedimentary DNA concentration. Newer PCR-reduced metagenomic approaches, such as shotgun sequencing targeting a broad spectrum of taxa from the full tree of life, now allow for a less biased assessment of the taxonomic composition of past ecosystems with a single sequencing run (34, 49, 50).

In a pioneering study (14), leveraged land-plant ancient DNA from six globally distributed marine sediment cores covering the Last Glacial-Holocene transition as a proxy for the share, burial rate (BR), preservation, and composition of terrigenous organic matter. They found that spatial and temporal plant sedaDNA records mainly reflect the vegetation dynamics of nearby continents. By establishing a calculation to relate plant sedaDNA concentrations to bulk accumulation rates and water content, they were even able to estimate the BR of terrestrial plant material derived by sedaDNA. They found a substantial proportion of plant DNA derived from riverine woodland taxa (Fig. 1), opening new avenues to explore the dynamic riverine woodland contribution to marine sedimentary organic matter in the context of global climate and cryospheric change.

## Deglacial riverine woodland expansion and enhanced terrestrial carbon burial in marine sediments

**Figure 2:**
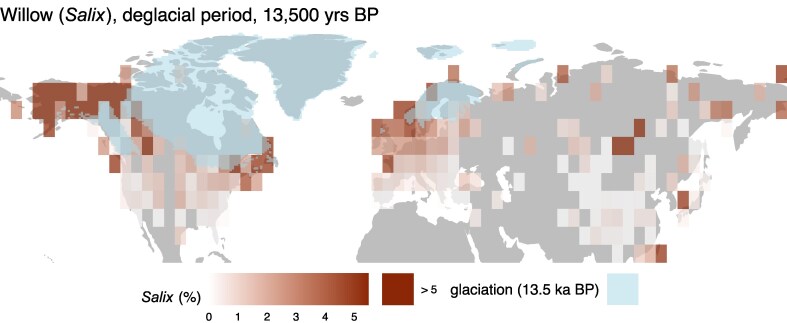
Willow percentage in deglacial (13,500 years BP) pollen records in the extratropical northern hemisphere (53). High willow abundances is found in regions characterized by, compared to today, expanded riverine wetland related to extended runoff from glacier and ice-sheet decay (54–58).

How ongoing global warming ultimately impacts the burial of terrestrial organic matter in marine sediments can to some extent be inferred by taking the Glacial-Holocene transition as an analogy. At that time warming forced ice sheets and glaciers to decay, peaking between 16 and 8 ka (Fig. 1A, B) (33). This resulted in an enhanced freshwater runoff (Fig. 1B) which not only expanded the totally flooded area but also increased bar turnover and floodplain habitat renewal, which favors pioneer riparian vegetation. Greater nutrient supply, resulting from the exposure of fresh mineral surfaces undergoing weathering processes (51), further supported their productivity. Widespread riparian wetland colonization during this period is indicated by the on average 3-fold increase of willow (*Salix*) in pollen time series from continents of the mid-to-high northern latitudes compared with the period before and after the deglacial peak period (Fig. 1C). Mapped *Salix* pollen percentages for 13.5 ka (52) trace the expanded riverine woodlands in the vicinity of the decaying northern hemisphere ice sheets and glaciers (Fig. 2). This also fits the findings from plant metabarcoding analyses of lake sediments from the northern high latitude, where willow shows higher proportions during the Late Glacial period compared with Holocene records (eg 59–61).

Riverine woody taxa contribute significantly to land plant aDNA recorded in marine sediments (Fig. 1, 14; Supplementary Table S2). For example, on average 49% of all land plant DNA sequences are derived from willow in four sediment records from the coastal and shelf areas in the Northwest Pacific and show a characteristic temporal pattern. The *Salix* DNA peak in these marine records from 15 to 13 ka BP (Bølling–Allerød warming period, Fig. 1C) is contemporary with the *Salix* expansion on land as inferred from northern hemisphere pollen records (Fig. 1C). Even more, not only the relative share of *Salix* DNA reads was increased but also the absolute *Salix* DNA BR was elevated during this period (Fig. 1D and E) which is in agreement of higher lignin (27) and total OC BR (14).

Availability of siliciclastic material is considered a limiting factor for the formation of organo-mineral complexes (44, 62) that protect organic matter, including extracellular DNA, against degradation (45, 63). The deglaciation uncovered large amounts of unconsolidated fine-grained siliciclastic material, which was eroded by and transported through rivers and as such contributed to high burial efficiency during the deglacial. Consistent with a preservation component, sedimentological analyses indicate higher sedimentation rates during deglacial runoff phases in the North Pacific (64), and higher plant sedaDNA read length, suggesting reduced degradation (14). In addition, land-plant DNA BR closely tracks lignin accumulation in a proximal Bering Sea core, indicating coherent behavior across independent vascular plant proxies (14, 27). It may be assumed that longer residence times of organic matter in floodplains lead to a preferential survival of matter that became stabilized through metal complexing and mineral protection (7), thereby increasing the likelihood that this stabilized fraction is subsequently remobilized and delivered to marine depocenters where it can be efficiently buried.

Sea-level changes may also have supported elevated terrestrial organic matter burial rates during the deglaciation. During the Glacial, the exposed shelves were likely widely covered by riverine lowlands with abundant *Salix*, for example, the Yukon delta. The permafrost conditions and the supply of loess supported the accumulation of stabilized organic matter eg in Yedoma permafrost soils (65). This permafrost soil organic matter became partly mobilized during shelf flooding at the beginning of the Bølling–Allerød (25), when global sea level has risen by up to 20 m over the short time of ∼500 years (66). River networks can transport and integrate environmental DNA downstream on suspended particulate matter, so nearshore and shelf sediments may archive a catchment-integrated ecosystem signal (67). The close co-occurrence of *Salix* peaks in terrestrial pollen records (Fig. 1C) and in our marine sedaDNA records (Fig. 1D) therefore suggests that the marine signal predominantly reflects contemporaneous, watershed-scale riparian expansion during the deglaciation rather than being dominated by remobilization of older lowstand shelf deposits—while still being biased toward lowland floodplains and newly inundated shelf plains that were preferentially connected. Together, our results suggest that *Salix,* representing a mainly riparian woodland source, was a key taxon for carbon burial in high-latitude marine sediments during the deglacial and that the related climate feedbacks were potentially of global relevance.

Some tropical regions experienced high runoff during the deglacial period, particularly due to the melting of mountain glaciers (68). The portion of Rhizophoraceae sedaDNA increased values during the deglacial period in a marine sediment record from the tropical western Atlantic, in agreement with a mangrove biomarker record (Fig. 1F) (Taraxerol/n-C_28_-ol, 36), which likely reflects increased mangrove extent of the Orinoco and other estuaries in the region.

## Does the order of magnitude in riverine woodland contribution (change) to marine carbon burial matters to global carbon cycle

Despite their limited spatial extent (global floodplain surface area ≈1.4 × 10^6^ km^2^ (69); mangroves ≈138,000 km^2^ (70); together ∼1% of land area), riverine woodlands can be disproportionately represented in land-to-ocean carbon transfer, consistent with our sedaDNA evidence for strong riparian woody-taxon contributions to terrigenous organic-matter burial. Rivers export ∼0.18 ± 0.04 Pg C year^−1^ of particulate organic carbon (POC) globally (6), and revised “turbidity-current pump” budgets imply terrestrial OC burial in marine sediments of 62–90 Mt C year^−1^ during the Holocene and 130–175 Mt C year^−1^ during the glacial (71). Using *Salix*—a dominant riparian woody taxon in northern hemisphere temperate and high-latitudes—as a conservative tracer, our *Salix* sedaDNA fractions recalculated with a 10 ka threshold (Holocene mean 34.2% of land-plant reads; pre-10 ka (glacial) mean 55.6%), and propagating uncertainty in (i) biospheric versus petrogenic riverine POC (median≈0.76; 95% range 0.62–0.87; 3) and (ii) aquatic versus terrestrial contributions to exported particulate organic matter (POM) (median aquatic fraction ≈0.49; 95% range 0.17–0.68; 72, 73), yields a riverine woodland-sourced marine terrestrial-OC burial flux of 10.0 (5.7–17.4) Mt C year^−1^ in the Holocene and 32.8 (19.2–55.3) Mt C year^−1^ during the glacial. This corresponds to a 3.28× (2.40–4.55×) increase, ie +22.7 (12.7–39.7) Mt C year^−1^ relative to Holocene conditions. Expressed as a fraction of total terrestrial marine OC burial, this implies riverine woodland-sources contribute 0.132 (0.078–0.223) during the Holocene versus 0.215 (0.128–0.357) during the glacial, ie ∼13% (8–22%) versus ∼22% (13–36%)—about ∼13–22× higher than expected from ∼1% areal extent (first order).

To connect the marine-only estimate to the broader land–ocean aquatic continuum (inland waters + estuaries/tidal wetlands + shelves + open ocean, 74), applying the same proportional scaling to the preindustrial long-term sediment-burial sink of ∼0.55 Pg C year^−1^, gives 0.072 (0.038–0.130) Pg C year^−1^ Holocene-like versus 0.118 (0.064–0.208) Pg C year^−1^ pre-10 ka, ie an excess of 0.046 (0.021–0.086) Pg C year^−1^. Integrated over 1,000 years, that is 46 (22–86) Pg C of additional burial, equivalent to a mass-balance upper bound of ∼21 (10–40) ppm CO_2_ (1 ppm ≈ 2.12 PgC), while acknowledging that air–sea partitioning and carbonate/weathering compensation, among others, can attenuate and time-lag the realized atmospheric response. For context, modern anthropogenic CO_2_ emissions are ∼11 Pg C year^−1^ (75), so this riparian-woody burial shift is small on annual scales but can become nontrivial when integrated over millennia, ie on glacial–interglacial timescales.

## Land use impact on riverine woodlands and its consequences for carbon burial

**Figure 3:**
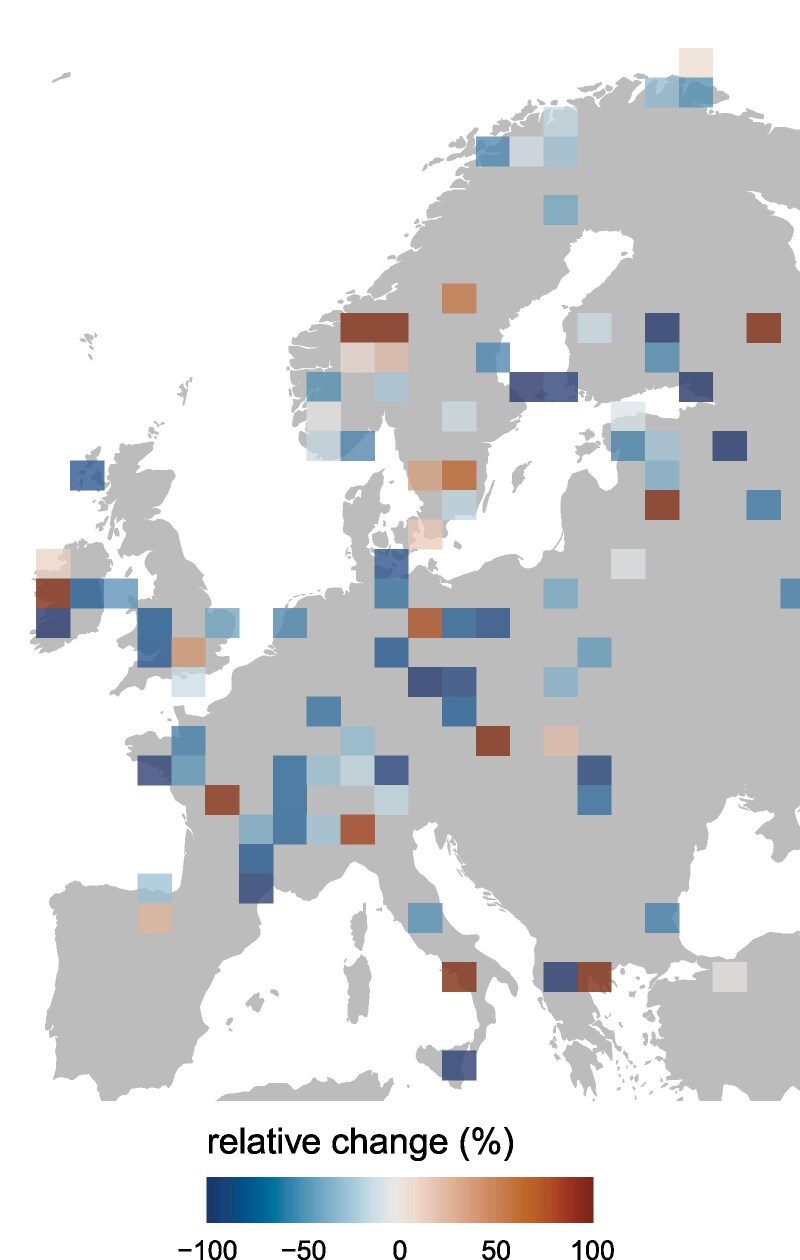
Riverine woodland loss (blue) and gain (red) during the last millennium known as a period of land use intensification. Indicated is the relative coverage change of willow between 1100 CE and 1900 CE as percentage of the willow coverage at 1100 CE. Taxa coverages are based on quantitative reconstruction leveraging pollen data (79) (only boxes >1.5% coverage were included to increase signal-to-noise ratio. A total of 426 pollen records contributed to the plot.).

Land-use changes have reduced riverine woodlands by about 21% since 1700 AD (22) and by that perturbed the carbon cycle along the land–ocean aquatic continuum (74) for thousands of years (76). While recently a good understanding of land use impact on broad-scale land cover was gained from synthesizing pollen evidence eg for Europe (22) and China (77, 78), tracing widespread human impact history specifically on riverine wetlands is missing. Substantial woody wetland loss in Europe since the medieval may be suggested by reconstructed *Salix* cover decline, particularly in Central Europe which experienced agricultural intensification in this period (Fig. 3). This confirms documented wetland loss of 25–70% in these areas during the last three centuries (22).

Riverine forest decline is not only a result of deforestation but also of river engineering. Channel straightening and building of dykes for flood protection results in ground water level lowering, which often leads to the drying of wetlands in the floodplain (80). Also, constraining the river to its bed and preventing meandering and other lateral river movement through the floodplain, disconnects the river from the floodplain limit and also hinders floodplain habitat renewal and wetland vegetation succession. This is in line with the finding that the portion of remaining active floodplains of rivers in these areas is small, eg 69% of Elbe and 59% of Rhine river floodplains are not anymore regularly flooding (81).

Land use resulted in a significant loss of riverine wetlands worldwide (22). In China, the expansion of rice paddies since the Han Dynasty (∼2,000 years ago) substantially altered the riverine wetlands (82), which supported the erosion of soils as inferred from biogeochemical analyses of the Yangtze estuary sediment (83). About 25% of riverine wetlands were lost in China during the last 100 years (84).

Straightening rivers and draining riverine wetland essentially eliminates all lateral sediment transfer (13). This lateral sediment transfer, however, is an important mechanism for transferring stabilized soil and sedimentary organic matter into the active channel and subsequently into long-term marine sinks (85). Reduced sediment supply downstream of dams has also been linked to channel incision and constrained river migration overall reducing bank renewal (86). Still the effect of human floodplain interventions on organic matter fluxes remains largely unquantified in many of the world's river systems but are expected to be significant (13, 87).

## Conclusions and outlook

The analyses of ancient DNA contained in marine sediments can quantitatively identify the taxonomic composition of source organisms contributing to organic matter in marine sediments. Together with the traditional biogeochemical and paleontological methods, this provides new opportunities to specify the source areas, timing and type of ecosystem that mostly contribute to the sedimentary carbon sink.

We compiled paleogenomic evidence from the northern North Pacific and off-Tobago that indicates that riverine woodland taxa form a substantial portion of the sedimentary plant DNA, despite covering only a few percent of the land area. This applies in particular to the deglacial period when extended meltwater run-off from decaying ice sheets and glaciers led to the expansion of riverine woodlands and estuary mangroves.

High availability of siliciclastic material and generally high sedimentation rates likely enhanced burial efficiency. To extrapolate these findings globally, further analysis of sedaDNA records from other regions is needed. Our results also indicate key taxa, namely willow (*Salix*) in the mid-to-high northern hemisphere and Rhizophoraceae in the tropics, which strongly contribute to or even absolutely dominate land carbon burial. The high productivity of these riverine and mangrove taxa, their ability to grow in flowing and under low oxygen conditions as well as their mechanisms to resist decay contributed to land-to-ocean transport and burial efficacy. However, a systematic assessment of which plant traits enhance the carbon sink function of land plant matter in (marine) sediments is needed.

In future, shotgun metagenomics could ultimately support first-order, source-resolved organic-carbon budgeting by translating sedimentary DNA concentrations into biomass/OC estimates, but only if taxon-specific biases in extraction efficiency, preservation, and read recovery are explicitly accounted for (88, 89) and DNA signals are anchored with organismal DNA-content and carbon-mass information (90) while considering taxon-specific mineral adsorption and physical shielding processes that stabilize DNA and associated organic matter over long timescales (63).

The studied records have a temporal resolution of multimillennia and originate from marine regions with sediment supply from watersheds with near-natural vegetation, which does not allow for detection and quantification of recent changes due to intensified human impact. However, the increase in carbon burial in subantarctic fjords with recent glacier retreat (91) generally indicates that in near-natural environments the negative feedback loop works also on decadal time scales.

**Figure 4:**
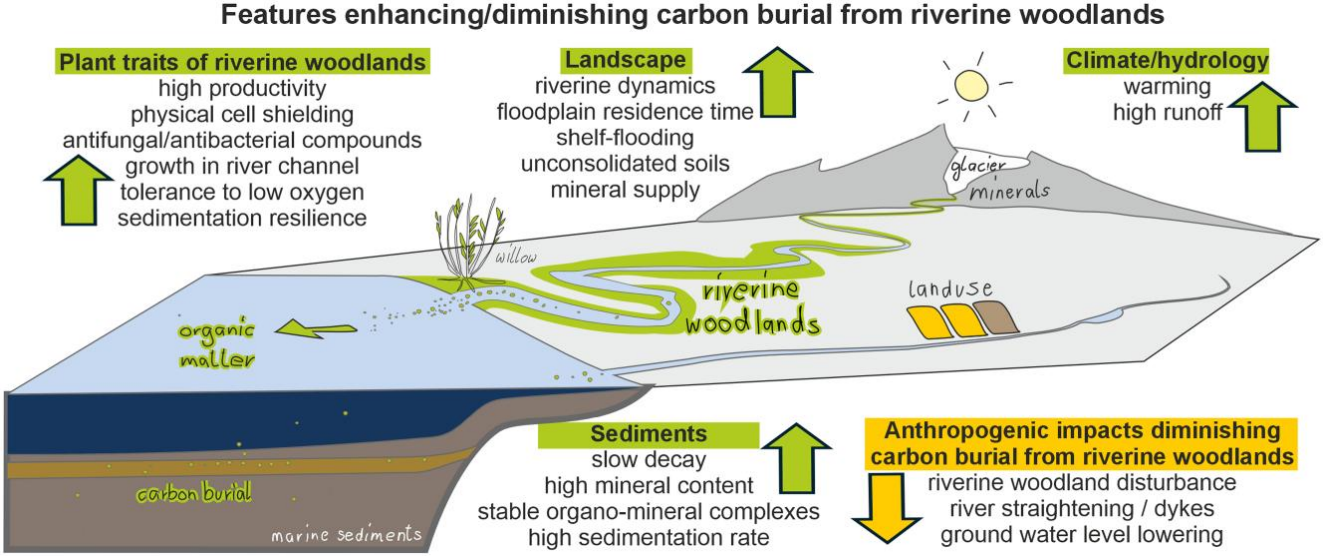
Illustration of the plant traits, landscape characteristics, climate conditions, and sediment features that support the burial of organic matter derived riverine woodland in marine sediments under natural conditions. Anthropogenic disturbance of riverine woodland (yellow) likely diminishes carbon burial.

Future riverine woodland change may vary regionally due to complex interactions between changing climate and human activity (Fig. 4) (21, 92). Ultimately, predictions can only be implemented using Earth system models. However, state-of-the-art models miss key components and functions (92) such as riverine wetlands and floodplains in general and their function as carbon source, carbon reactor, and modulator of carbon burial. This is also because ecosystem service assessments of riverine woodlands often overlook the importance of these systems for the long-term sedimentary carbon sink (93): riparian woodlands are directly situated at the link between the short-term biological carbon cycle (photosynthetic carbon fixation) and the long-term geological carbon cycle (marine sediment sinks). Even more, optimization tools to identify new areas for protection are established separately for terrestrial (94) and marine areas, which does not allow optimization for the ecosystem service of land-to-ocean carbon transfer. And as such, the potential of managing riverine woodlands as a natural carbon capture and storage potential was not yet systematically assessed. We therefore identify these systems as key future research areas to quantify human impacts and assess the potential of riverine woodlands as natural carbon capture and storage locations.

## Supplementary Material

pgag229_Supplementary_Data
