# Supplementary material for: Riverine woodlands as a dynamic source of the marine sedimentary carbon sink
Source: PNAS Nexus. 2026 Jun 25;5(7):pgag229. doi: 10.1093/pnasnexus/pgag229 (PMC13347976; doi:10.1093/pnasnexus/pgag229)
Supplement: pgag229_Supplementary_Data [file pgag229_supplementary_data.docx]

**Supplementary Material**

**Supplementary Text 1 First order estimation of Riverine woodland‑sourced long‑term burial)**

We estimated the long‑term **Riverine woodland‑sourced terrestrial OC burial flux** by *scaling published global terrestrial OC burial budgets* with three independent fractions that progressively restrict the signal to (i) **biospheric** (non‑petrogenic) river POC, (ii) **non‑aquatic** (non‑autochthonous) material, and (iii) the **Salix share within land‑plant sedaDNA** (this study) as tracer of riverine woodland share. As global endmembers for terrestrial OC burial in marine sediments, we used the revised “turbidity‑current pump”/marine burial budgets that imply **62–90 Mt C yr⁻¹** during interglacial highstands and **130–175 Mt C yr⁻¹** during glacial lowstands.

In compact form, the scaling can be written as:
 **F_Salix = B_marine × f_bio × f_non‑aq × f_Salix**,
 where *B_marine* is terrestrial OC burial in marine sediments, *f_bio* is the biospheric fraction of exported POC, *f_non‑aq* is the fraction not attributable to aquatic biomass production in rivers, and *f_Salix* is the mean Salix fraction of land‑plant sedaDNA for each time slice (Holocene ≤10 ka; pre‑10 ka).

How the fractions and uncertainties were defined

**Biospheric vs petrogenic partitioning (f_bio).** We derived the biospheric fraction from published global export estimates of **biospheric POC (157 +74/−50 Mt C yr⁻¹)** and **petrogenic POC (43 +61/−25 Mt C yr⁻¹)** (Galy et al., 2015). In each uncertainty draw, *f_bio* is computed from the sampled biospheric and petrogenic contributions (so that higher petrogenic export reduces the biospheric fraction available for atmospheric CO₂ sequestration).

**Aquatic vs non‑aquatic contribution (f_non‑aq).** To constrain the fraction of riverine POC that can be attributed to *non‑aquatic* sources (vascular plants/soils), we bracketed plausible endmembers using (1) a pan‑Arctic source‑mixing constraint showing that **aquatic biomass contributes a median ~51% of pan‑Arctic POC flux (5–95% range ~39–60%)** and (2) a temperate‑river compilation indicating **annual phytoplankton contributions spanning ~5–80%**. We treated these constraints as equally plausible bracketing distributions to avoid relying on a single climatic setting, and set *f_non‑aq* as the complement of the sampled aquatic fraction.

**Salix fraction (f_Salix; this study).** For each sample, Salix percentage was computed as **Salix reads / total land‑plant reads**. Period means for the Holocene (≤10 ka) and pre‑10 ka were recomputed using the 10 ka threshold, and uncertainty in the mean was quantified by bootstrap resampling of samples within each time slice.

Uncertainty propagation and reporting

All uncertain inputs (marine burial budgets, biospheric/petrogenic partitioning, aquatic fraction, and Salix fraction) were propagated using a Monte‑Carlo approach (large‑N random sampling of the input distributions). For each realization, we computed Holocene and pre‑10 ka **F_Salix**, and then calculated the **ratio** (pre‑10 ka / Holocene) and **difference** (pre‑10 ka − Holocene) draw‑by‑draw. We report medians and 95% uncertainty intervals (2.5th–97.5th percentiles).

LOAC scaling and ppm conversion (context only)

To provide a **land–ocean aquatic continuum (LOAC)** context beyond the marine‑only burial budgets, we also applied the composite fraction *(f_bio × f_non‑aq × f_Salix)* to the pre‑industrial **total long‑term sediment burial across inland waters + estuaries/tidal wetlands + shelves + open ocean**, reported as **0.55 Pg C yr⁻¹** in the Regnier et al. (2022) supplementary flux synthesis.

Carbon mass was converted to an atmospheric CO₂ **mass‑equivalent** using **1 ppm ≈ 2.12 PgC**. These ppm values are upper‑bound mass equivalents (air–sea partitioning and carbonate/weathering compensation are not included).

**Supplementary Table S1. Inputs (with uncertainty ranges) used for estimating Riverine woodland-sourced terrestrial OC burial fluxes**

| **Parameter** | **Symbol** | **Central / range** | **Uncertainty type** | **Units** | **Source / note** | **Reference** |
| --- | --- | --- | --- | --- | --- | --- |
| Marine burial flux of terrestrial OC (interglacial highstand) | B_marine_highstand | 62–90 | Uniform (min–max) | Mt C yr⁻¹ | Hilton & West review, Table 1 ("This review"; terrestrial total POC burial flux at present-day highstand) | Talling et al. (2024) |
| Marine burial flux of terrestrial OC (glacial lowstand) | B_marine_lowstand | 130–175 | Uniform (min–max) | Mt C yr⁻¹ | Hilton & West review, Table 1 ("This review"; terrestrial total POC burial flux at glacial lowstand) | Talling et al. (2024) |
| Riverine biospheric POC export (terrestrial biosphere) to ocean | F_bio | 157 (+74/−50) ⇒ 107–231 | Triangular (min, mode, max) = (107,157,231) | Mt C yr⁻¹ | Galy et al. 2015 (Nature): global erosion-controlled biospheric POC export | Galy et al. (2015) |
| Riverine petrogenic POC export to ocean | F_petro | 43 (+61/−25) ⇒ 18–104 | Triangular (min, mode, max) = (18,43,104) | Mt C yr⁻¹ | Galy et al. 2015 (Nature): global erosion-controlled petrogenic POC export | Galy et al. (2015) |
| Biospheric fraction of riverine POC (computed) | f_bio = F_bio/(F_bio+F_petro) | median 0.76 (0.62–0.87) | Derived from triangular F_bio and F_petro | — | Computed in Monte Carlo from Galy et al. 2015 flux uncertainties | Galy et al. (2015) |
| Autochthonous aquatic fraction of exported POM/POC (Arctic constraint) | f_aq_Arctic | median 0.51 (0.39–0.60) | Triangular (min, mode, max) = (0.39,0.51,0.60) | fraction | PNAS 2023 pan-Arctic mixing model (aquatic biomass share of annual POM flux) | Behnke et al. (2023) |
| Autochthonous aquatic fraction of exported POM/POC (temperate constraint) | f_aq_temp | 0.05–0.80 (annual-scale phytoplankton range across rivers) | Triangular (min, mode, max) = (0.05,0.45,0.80) | fraction | Ferchiche et al. 2025 (Biogeosciences): phytoplankton contribution range 5–80% at annual scale | Ferchiche et al. (2025) |
| Autochthonous aquatic fraction used in Monte Carlo | f_aq | median 0.49 (0.17–0.68) | Mixture: 50% Tri(0.39,0.51,0.60) + 50% Tri(0.05,0.45,0.80) | fraction | Combines Arctic + temperate constraints (equal weight, to bracket plausible modern range) |  |
| Non-aquatic (terrestrial) fraction of biospheric POC | f_non_aq = 1 − f_aq | median 0.51 (0.32–0.83) | Derived from f_aq | fraction | Computed |  |
| Salix fraction of land-plant sedaDNA (Holocene, ≤10 ka) | f_Salix_H | mean 0.342; bootstrap 95% CI 0.281–0.405 | Empirical bootstrap distribution of the mean (n=50,000) | fraction | This study (NW Pacific shelf cores; Salix reads / land-plant reads) | This study |
| Salix fraction of land-plant sedaDNA (pre-10 ka, >10 ka) | f_Salix_G | mean 0.556; bootstrap 95% CI 0.509–0.595 | Empirical bootstrap distribution of the mean (n=50,000) | fraction | This study (NW Pacific shelf cores; Salix reads / land-plant reads) | This study |
| Pre-industrial LOAC long-term sediment burial (lakes + estuaries/tidal wetlands + shelf + open ocean) | B_LOAC | 0.55 | Normal; 2σ≈0.18 from quadrature of Table S1 component 2σ | Pg C yr⁻¹ | Regnier et al. 2022 Supplementary Table S1 (F°IS + (F°WS+F°ES) + F°CS + F°OS) | Regnier et al. (2022) |
| Potential additional floodplain burial (not included in LOAC burial) | B_floodplain | 0.19 ± 0.13 | Normal; treated as ±2σ | Pg C yr⁻¹ | Regnier et al. 2022 Supplementary text (floodplain burial could reach 0.19 ± 0.13 PgC yr−1) | Regnier et al. (2022) |
| Atmospheric conversion | k_ppm | 1 ppm CO₂ ≈ 2.12 Pg C | Constant | Pg C ppm⁻¹ | Stoichiometric conversion (atmospheric mass; used for mass-equivalent upper bound) |  |

**References**

Talling, P. J., Hage, S., Baker, M. L., Bianchi, T. S., Hilton, R. G., & Maier, K. L. (2024). The Global Turbidity Current Pump and Its Implications for Organic Carbon Cycling. Annual Review of Marine Science, 16, 105-133. https://doi.org/10.1146/annurev-marine-032223-103626.

Galy, V., Peucker-Ehrenbrink, B., & Eglinton, T. (2015). Global carbon export from the terrestrial biosphere controlled by erosion. Nature, 521(7551), 204-207. https://doi.org/10.1038/nature14400.

Behnke, M. I., Tank, S. E., McClelland, J. W., Holmes, R. M., Haghipour, N., Eglinton, T. I., ... & Spencer, R. G. M. (2023). Aquatic biomass is a major source to particulate organic matter export in large Arctic rivers. Proceedings of the National Academy of Sciences of the United States of America, 120(12), e2209883120. https://doi.org/10.1073/pnas.2209883120.

Ferchiche, F., Liénart, C., Charlier, K., Deborde, J., Giraud, M., Kerhervé, P., Polsenaere, P., & Savoye, N. (2025). Toward a typology of river functioning: a comprehensive study of the particulate organic matter composition at the multi-river scale. Biogeosciences, 22, 7363-7401. https://doi.org/10.5194/bg-22-7363-2025.

Regnier, P., Resplandy, L., Najjar, R. G., & Ciais, P. (2022). The land-to-ocean loops of the global carbon cycle. Nature, 603(7901), 401-410. https://doi.org/10.1038/s41586-021-04339-9.

**
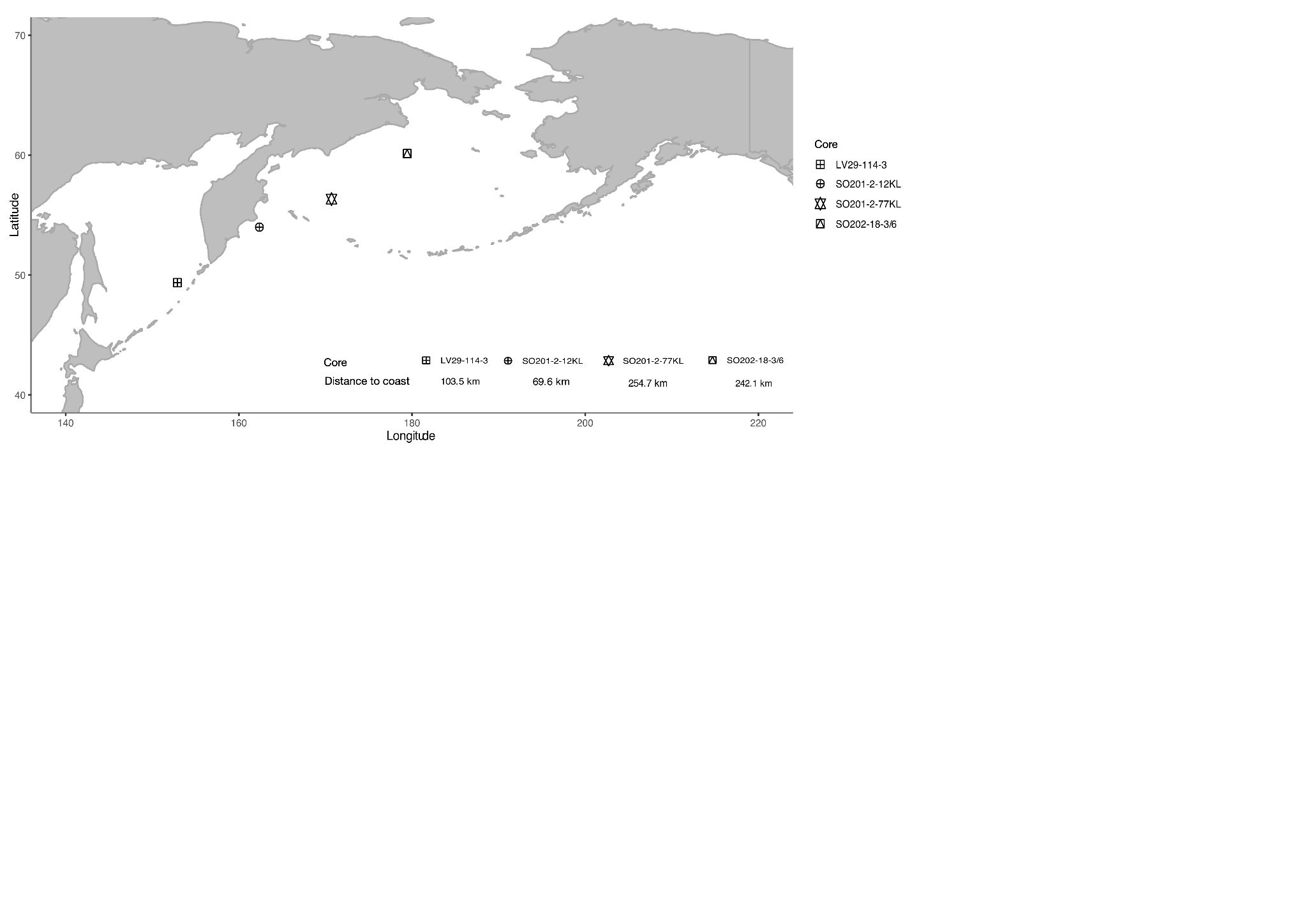
**

**Figure S1:** Map of sediment core locations in the North Pacific and Bering Sea. Symbols indicate the positions of the four marine records used in this study: LV29-114-3 (Sea of Okhotsk), SO201-2-12KL (Off Kamchatka), SO201-2-77KL (Bering Sea), and SO202-18-3/6 (Bering Sea Shelf). The legend provides the calculated shortest distance to the modern coastline for each site, ranging from 69.6 km (SO201-2-12KL) to 254.7 km (SO201-2-77KL).

**Supplementary Table S2. Sequencing data availability of marine sediment cores used in this study.**

| **Core** | **Accession Number** | **Published in** | **Code availabilty** |
| --- | --- | --- | --- |
| SO201-2-12KL | PRJEB46821 | Zimmermann et al., 2023 | <https://github.com/JoFrieWeiss/Riparian-Woodland> |
| SO201-2-77KL | PRJEB46821 | Buchwald et al., 2024 | <https://github.com/JoFrieWeiss/Riparian-Woodland>) |
| M78/1-235-1 | PRJEB74341 | Herzschuh & Weiß et al., 2025 | <https://github.com/JoFrieWeiss/Riparian-Woodland>) |
| SO202-18-3/6 | PRJEB108548 | Weiß et al., 2026 (preprint) | <https://github.com/JoFrieWeiss/Riparian-Woodland>) |
| LV29-114-3 | PRJEB108549 | Weiß et al., 2026 (preprint) | <https://github.com/JoFrieWeiss/Riparian-Woodland>) |
